# Single Nucleotide Polymorphisms of *EXOC1*, *BCL2*, *CCAT2*, and *CARD8* Genes and Susceptibility to Cervical Cancer in the Northern Chinese Han Population

**DOI:** 10.3389/fonc.2022.878529

**Published:** 2022-06-24

**Authors:** Yanan Feng, Zhenzhen Wang, Manning Zhu, Songxue Li, Shuang Dong, Liping Gong, Xiaoying Li, Shuang Zhang, Tianshuang Jia, Xianchao Kong, Jiawei Tian, Litao Sun

**Affiliations:** ^1^Cancer Center, Department of Ultrasound Medicine, Zhejiang Provincial People’s Hospital, Hangzhou, China; ^2^Department of Ultrasound, The Second Affiliated Hospital of Harbin Medical University, Harbin, China; ^3^Department of Obstetrics and Gynecology, The Second Affiliated Hospital of Harbin Medical University, Harbin, China

**Keywords:** cervical cancer, different genes, SNP, susceptibility, multiplex polymerase chain reaction

## Abstract

Cervical cancer (CC) is one of the main malignant tumors that threaten the health and lives of women around the world, and its morbidity and mortality rate ranks fourth. At present, most studies on the genetic background of CC focus on genetic polymorphisms. Single nucleotide polymorphisms (SNPs) are considered clinically as potential diagnostic and therapeutic biomarkers for a variety of tumors. Therefore, we aimed to explore the association between SNPs in different genes (*EXOC1* gene, *BCL2* gene, *CCAT2* gene and *CARD8* gene) and susceptibility to CC. This study is a case-control study based on women in northern Chinese, which included 492 women with CC and 510 healthy women. This study used multiplex PCR combined with next-generation sequencing to genotype the selected SNPs (rs13117307(C/T) in *EXOC1* gene, rs2279115(C/A) in *BCL2* gene, rs6983267(G/T) in *CCAT2* gene and rs7248320(G/A) in *CARD8* gene). The results of the study showed that there was no significant association between the four SNPs and the susceptibility to CC. However, in further stratified analysis, we found that rs13117307 and rs2279115 were significantly related to squamous cell carcinoma antigen (SCC-Ag) levels in women with CC, and rs6983267 was significantly related to the menopausal status of women with CC. Specifically, alleles T of rs13117307 and genoytpe AA of rs2279115 when SCC-Ag is greater than 1.5 ng/ml increase the risk of CC. The genotype TG/TG+TT of rs6983267 increases the risk of CC in premenopausal women. In conclusion, although we did not directly find a significant correlation between four SNPs, rs13117307 in EXOC1 gene,rs2279115 in BCL2 gene, rs6983267 in CCAT2 gene and rs7248320 in CARD8 gene, and CC susceptibility, we found that SNPs rs13117307, rs2279115, rs6983267 were associated with the clinical characteristics of several patients' CC patients. Therefore, this study provides us with new ideas for understanding CC and the diagnosis and treatment of CC in the future.

## Introduction

Cervical cancer (CC) is one of the common malignant tumors in gynecology. It has a high morbidity and mortality rate worldwide, which seriously threatens the life and health of women. There are about 604000 new CC patients every year, of which 75-80% occur in developing countries (1), which brings a heavy economic burden to society and families (2). With the popularization of cervical cytology screening, the use of HPV vaccines and the improvement of protection awareness, the morbidity and mortality rate of CC has decreased, but CC is still a serious public health problem (3, 4). Early effective prevention and diagnosis are particularly important for the treatment of CC.

CC usually occurs at the junction of cervical squamous column, from atypical hyperplasia of cervical epithelium to intraepithelial carcinoma in situ, to invasive carcinoma, and finally to metastatic carcinoma. This process may take several years or more than ten years. Current research shows that viral infection, premature or disordered sexual life, early pregnancy, prolificacy and eating habits are all related to the increased risk of CC. Among them, HPV infection is recognized as a high risk factor for CC (5, 6). Studies have confirmed that the incidence of female first-degree relatives suffering from CC is higher than that of the general population, suggesting that genetic factors play an important role in the pathogenesis of CC (7–9). This fact encourages some research groups to look for susceptibility genes for CC. SNP is the most common genetic variation in human genes and is considered to be a new generation of genetic markers. Clinically, SNPs can be used as potential biomarkers for diagnosis and treatment of various tumors (10, 11). Tumor necrosis factor -α Promoter gene (12), polymerase II polypeptide E (POLR2E) (13), ganglioside biosynthesis gene (6), methylenetetrahydrofolate reductase (MTHFR) gene (14) polymorphisms were associated with CC.

The exocyst complex component 1 (EXCO1) protein encoded by the *EXCO1* gene is one of the components of the exocyst complex (EXCO1-EXCO8). The complex promotes the regulation of cellular membrane exocytosis, secretion of the cell membrane and participate in cellular migration and secretion, and vesicle transport (15–18). Recently, some evidence suggests that the exocyst complex has an important role in the occurrence and development of various cancers (19). Apoptosis is an important mechanism for maintaining cell/tissue balance. Abnormal cell apoptosis is one of the important pathological basis of tumor formation, and it directly participates in the occurrence and development of tumors. The anti-apoptotic B cell lymphoma 2 (BCL2) gene is located on chromosome 18q21.3, encoding BCL2 protein, which is a typical apoptosis regulator (20, 21). BCL2 is found to be highly expressed in a variety of tumors, such as prostate cancer, non-small cell lung cancer, chronic lymphocytic leukemia, and diffuse large B-cell lymphoma (22–26). Caspase recruitment domain family member 8 (CARD8), also known as TUCAN, is a member of the amino acid protease recruitment domain family, which affects protein-protein interaction, apoptosis, caspase-1 activation, IL-1 and IL-8 activation and activation kappa-B (NFkB) (27, 28). NFkB is a key regulator of gene transcription and tumorigenesis. Colon Cancer-Associated Transcript 2 (CCAT2) long non-coding RNA is a transcript containing rs6983267 SNP (29), which can up-regulate MYC through TCF7L2-mediated transcription. Previous studies have shown that the rs6983267 SNP located in the MYC enhancer region is related to the susceptibility of various cancers, including prostate cancer, colorectal adenoma and cancer, thyroid cancer, endometrial carcinoma, and ovarian cancer (30–34).

However, report on the relationship between *EXOC1*, *BCL2*, *CARD8*, *CCAT2* gene and CC is rarely. Therefore, we aimed to explore the possible effect of *EXOC1*, *BCL2*, *CARD8*, *CCAT2* gene polymorphisms on the development of CC in the northern Chinese population.

## Material and Methods

### Study Subjects

This case-control study was recruited 492 CC patients and 510 healthy controls, which is carried out in Department of Gynecology, the Second Affiliated Hospital of Harbin Medical University, Harbin, Heilongjiang Province, China. The cases are all patients with primary CC without any treatment, and their histopathology has been confirmed by the Pathology Department of the Second Affiliated Hospital of Harbin Medical University. The exclusion criteria for the patients are: 1) cervical benign lesions; 2) cervical benign tumor; 3) other cervical malignant tumors or combined with other tumors; 4) cervical cancer patients with a history of radiotherapy and chemotherapy before surgery. The entry criteria of the healthy control group: 1) TCT negative; 2) No history of tumor; 3) Choose the same hospital at the same time. Exclusion criteria for healthy control group: 1) history of gynecological diseases and gynecological surgery; 2) immunocompromised and immune diseases; 3) skin or genital warts; 4) history of other diseases and family tumors. All participants are biologically unrelated han Chinese female living in northern China, and they are all non-probabilistic continuous samples.

All participants signed informed consent, which were approved by the Ethics Committee of the Second Affiliated Hospital of Harbin Medical University.

### Selection of SNPs

Based on previous studies and combined with the characteristics of East Asian population in the dbSNP database, we selected 4 SNPs: rs13117307(C/T) in *EXOC1* gene, rs2279115(C/A) in *BCL2* gene, rs6983267(G/T) in *CCAT2* gene and rs7248320(G/A) in *CARD8* gene. The minor allele frequency of the four SNPs is shown as follows: rs13117307 P_T_ = 0.1042; rs2279115 P_A_ = 0.43; rs6983267 P_T_ = 0.6121; rs7248320 P_A_ = 0.6587. Of the SNPs, rs13117307 and rs6983267 was located in the intron region, rs2279115 was located in promoter region, and rs7248320 was located in the 2kb upstream.

### Extraction of DNA

When all subjects were admitted to the hospital, peripheral venous blood was collected, placed in a 2% EDTA-Na2 anticoagulation tube, and refrigerated at -80°C for DNA extraction. Genomic DNA of all subjects was extracted in strict accordance with the standard steps of the TIANamp Genomic DNA Kit (Tiangen Biotech, China). The four SNPs of *EXOC1*, *BCL2*, *CCAT2* and *CARD8* gene were genotyped by Shanghai BioWing Applied Biotechnology Company (http://www.biowing.com.cn) using the multiplex PCR method combined with next-generation sequencing methods. Primer 3 online software (Version 0.4.0, http://frodo.wi.mit.edu/) was used to design amplification primers for the four SNPs. The amplification primers are as follows: for rs13117307 sense primer ‘ACAGGTTAAAAGGTAGTTTTGTAG’ and anti-sense primer ‘AAATTAGTGTGTCATCCTTGCAC’; for rs2279115 sense primer ‘CCTTCATTTATCCAGCAGCTTTTC’ and anti-sense primer ‘CAGAAGTCCTGTGATGTTTTCC’; rs6983267 sense primer ‘AAGAGGTGTAGCCAGAGTTAATAC’ and anti-sense primer ‘CTGTATACACAGCCCAGTCTAAG’; rs7248320 sense primer ‘CGTGAGAAAACATCAAAGAAATCC’ and anti-sense primer ‘TGCAGACCTTATTTGAATTTTGTC’. The mixture of PCR products were purified by TIANgel Midi Purification Kit (Tiangen Biotech, China). The purified PCR products were then paired-end sequenced by Illumina HiSeq XTen platform according to the manufacturer’s instructions. The readings were aligned to the human reference genome reference using Burrows - Wheeler Aligner (BWA, v0.7.12) , and Samtools (v0.1.19) (35) was used for SNP calling and genotyping. Seventy-three samples were randomly selected for blind DNA replication to control the quality of genotyping.

### Statistical Analysis

Genotype distribution and allele frequencies were compared between groups using the *X^2^* test of independence with a 2 × 2 contingency. The SHEsis software (http://analysis.bio-x.cn/myAnalysis.php) (36, 37) was used to test the haplotype analysis between controls and CC group. The lowest frequency threshold for haplotype was 0.03. Under the appropriate conditions, the odds ratios (ORs) with corresponding 95% confidence intervals (CIs) were calculated and used as a measure of the association between the SNPs genotype and the phenotype of the disease. *P* < 0.05 was considered statistically significant. Multivariable logistic regression analysis (SPSS V21.0 for Windows) was used to assess the association of allele and genotype of SNPs with CC after adjustment for age, height and weight. Multiple comparisons were counteracted by using the Bonferroni correction to adjust the statistical significance level for possible statistical and clinical confounders.

## Results

### SNP Genotype and Quality

Linkage disequilibrium among the SNPs was tested using SHEsis software. All SNPs were found to be in Hardy - Weinberg equilibrium in controls (*p* > 0.05, **Table 1**). The genotype calling rate in quality control samples were 98.5%, suggesting the reliability of subsequent studies.

**Table 1:** Hardy-Weinberg equilibrium test results of target SNPs in controls.

SNP	Controls	
	X^2^	*p*
rs13117307	0.862	0.353
rs2279115	0.028	0.867
rs6983267	0.078	0.779
rs7248320	0.899	0.343

### Clinical Characteristics of the Study Population

The average age of CC patients was 49.245 ± 9.412 years, and the average age of healthy controls was 39.490 ± 8.227 years. There is a significant difference in age between CC patients and healthy controls (*p* < 0.001). The distribution of clinical features of CC patients are shown in **Table 2**.

**Table 2:** The distribution of clinical features of CC patients and controls.

Variables	CC case (n = 492)	Controls (n = 510)
Average Age (mean ± SD)	49.245 ± 9.412*	39.490 ± 8.227*
Menophania Age		
< 15y	228	
≥ 15y	236	
Missing data	28	
Amenorrhea		
Yes	220	
No	246	
Missing data	26	
SCC-Ag		
≤ 1.5ng/ml	213	
> 1.5ng/ml	245	
Missing data	34	
Tumor Size	140	
≤ 4cm	242	
> 4cm	110	
Missing data	142	

CC, Cervical Cancer; *Compared with the controls, there was a statistically significant difference.

### SNPs and CC Risk

The frequencies of genotypes and alleles in gene between CC patients and healthy controls were demonstrated in **Table 3**. There was no statistically significant difference in genotype and allele frequencies between CC patients and controls. After using logistic regression analysis to adjusted age, height and weight factors, there was no association between CC patients and control (*p* > 0.05, all). Similarly, no SNPs were found to be associated with CC risk after Bonferroni correction (*p* > 0.05, all)

**Table 3:** The frequencies distribution of genotypes and alleles of four SNPs in CC patients and healthy controls.

SNP	Control	CC	*p*	OR [95%CI]	p*	OR [95%CI] *
rs13117307						
CC	380	377				
TC	115	100	0.395	1.141 [0.842-1.545]	0.952	1.014 [0.638-1.613]
TT	12	13	0.829	0.916 [0.413-2.033]	0.181	2.402 [0.664-8.682]
TC+TT	127	113	0.463	1.115 [0.834-1.491]	0.561	1.140 [0.733-1.773]
C/T	875/139	854/126	0.576	1.077 [0.831-1.395]	0.348	1.207 [0.815-1.789]
rs2279115						
CC	200	203				
AC	237	225	0.624	1.069 [0.818-1.397]	0.796	0.946 [0.622-1.440]
AA	68	59	0.442	1.170 [0.784-1.745]	0.301	1.399 [0.740-2.647]
AC+AA	305	284	0.505	1.090 [0.846-1.405]	0.901	1.026 [0.689-1.527]
C/A	637/373	631/343	0.427	1.077 [0.897-1.294]	0.302	1.167 [0.871-1.563]
rs6983267						
GG	90	78				
TG	247	229	0.708	0.935 [0.657-1.330]	0.073	0.593 [0.336-1.049]
TT	161	174	0.244	0.802 [0.553-1.162]	0.143	0.633 [0.343-1.168]
TG+GG	408	403	0.441	0.877 [0.629-1.224]	0.066	0.608 [0.358-1.033]
G/T	427/569	385/577	0.201	0.889 [0.743-1.064]	0.172	0.819 [0.615-1.091]
rs7248320						
GG	64	57				
AG	246	230	0.812	0.953 [0.639-1.421]	0.373	1.343 [0.702-2.570]
AA	197	203	0.483	0.864 [0.575-1.299]	0.451	1.290 [0.665-2.505]
AG+AA	443	433	0.632	0.911 [0.623-1.333]	0.362	1.330 [0.720-2.456]
G/A	374/640	344/636	0.407	1.080 [0.900-1.297]	0.562	0.917 [0.685-1.229]

*Age, height and weight were adjusted.

### SNPs and CC-Related Risk Factors

For SNP rs13117307, we found that the allele C/T of patients with CC is significantly correlated with squamous cell carcinoma antigen (SCC-Ag), that is, women with CC with allele T have higher risk when SCC-Ag is greater than 1.5ng/ml (*p* = 0.029). But after adjusting for factors such as age, height, and weight, the correlation disappeared (*p_adj_* = 0.159). Moreover, when SCC-Ag is greater than 1.5 ng/ml, the significant correlation between rs2279115 and CC is observed (CC vs. AA: *p* = 0.025). But after adjusting for factors such as age, height, and weight, the correlation disappeared (*p_adj_* = 0.27). The genotype TG/TG+TT of rs6983267 can increase the risk of CC in premenopausal women (GG vs. TG: *p*_adj_ = 0.003, GG vs. TG+TT: *p*_adj =_ 0.023). After Bonferroni correction, compared with CC patients carrying the GG genotype, there was an association between CC women carrying the TG genotype and SNP rs6983267 (Bonferroni-corrected p = 0.009); however, the correlation did not exist in the comparative analysis of GG and TG+TT(Bonferroni-corrected p = 0.069). However, SNP rs7248320 was not significantly different from CC - related risk factors (**Table 4**).

**Table 4:** Genetypic association between four SNPs (rs13117307, rs2279115, rs6983267 and rs7248320) and CC-related risk factors.

SNP		Age		Menophania Age		Amenorrhea		SCC		Tumor Size	
		*p*	*P*^#^	*p*	p*	*p*	p*	*p*	p*	*p*	p*
rs13117307(C/T)	CC/TC	0.298	0.747	0.961	0.805	0.546	0.724	0.189	0.229	0.984	0.796
	CC/TT	0.144	0.217	0.429	0.206	0.226	0.147	0.081	0.425	0.551	0.999
	CC/TC+TT	0.157	0.583	0.842	0.57	0.346	0.476	0.074	0.178	0.848	0.633
	C/T	0.083	0.424	0.649	0.381	0.212	0.304	**0.029**	0.159	0.713	0.481
rs2279115(C/A)	CC/AC	0.743	0.692	0.734	0.429	0.902	0.805	0.764	0.437	0.532	0.212
	CC/AA	0.432	0.4	0.446	0.416	0.122	0.792	**0.025**	0.27	0.313	0.276
	CC/AC+AA	0.592	0.57	0.974	0.691	0.531	0.781	0.321	0.313	0.394	0.17
	C/A	0.462	0.44	0.670	0.867	0.216	0.741	0.068	0.237	0.304	0.196
rs6983267 (G/T)	GG/TG	0.446	0.493	0.347	0.145	0.124	**0.003^&^**	0.855	0.161	0.769	0.699
	GG/TT	0.531	0.587	0.569	0.323	0.577	0.264	0.458	0.542	0.585	0.504
	GG/TG+TT	0.86	0.808	0.402	0.183	0.521	**0.023**	0.643	0.271	0.668	0.429
	G/T	0.288	0.33	0.724	0.515	0.189	0.812	0.393	0.903	0.581	0.992
rs7248320(G/A)	GG/AG	0.232	0.223	0.47	0.308	0.279	0.533	0.569	0.968	0.32	0.804
	GG/AA	0.373	0.209	0.873	0.832	0.317	0.462	0.834	0.512	0.376	0.71
	GG/AG+AA	0.265	0.179	0.629	0.52	0.271	0.493	0.832	0.687	0.316	0.893
	G/A	0.633	0.334	0.808	0.629	0.483	0.453	0.478	0.335	0.541	0.554

SCC, squamous cell carcinoma antigen.

^#^Height and weight were adjusted. *Age, height and weight were adjusted.

p values < 0.05 are shown in bold.

^&^Indicates that the significance persists after Bonferroni’s adjustment.

### Haplotype Analysis

To further investigate the relationship between CC and haplotype distributions of *EXOC1*, *BCL2*, *CCAT2* and *CARD8* gene, we conducted haplotype analysis. However, there were no significant differences observed controls and patient groups (*p* > 0.05, data not shown).

## Discussion

CC is the fourth most common cancer in the world and the leading cause of cancer deaths in women worldwide (1). Persistent infection of carcinogenic human papilloma virus (HPV) and chronic inflammation are currently more recognized causes of CC. However, studies have shown that only a small percentage of women infected with HPV will eventually develop CC (38). Therefore, the development of CC is affected by many factors, among which genetic susceptibility cannot be ignored. Gene polymorphism is the simultaneous and frequent presence of two or more discontinuous variants or genotypes or alleles in a biological population, including DNA fragment length polymorphism, DNA repetitive sequence polymorphism and SNP. SNP is characterized by the most common, high density, representativeness and genetic stability. This study was a hospital-based case-control study. We observed the distribution of the genotypes, alleles and haplotypes of *EXOC1*, *BCL2*, *CCAT2* and *CARD8* gene in CC patients and healthy controls.

Multiple evidences indicate that CD8+ T cell-mediated immune response is very important in HPV infection and virus-related neoplasia (39, 40). EXCO1 protein is one of the components of the exocyst complex. The binding of the exocyst complex and NEF protein may play an important role in down regulating MHC-I coding genes and regulating T cell signaling pathways (41–43). In addition, the fusion of NEF mutation with HPV-16 type protein E7 induced an anti-E7 CD8+ cytotoxic T lymphocyte response, which is related to the protection of HPV-related tumors (44). The disruption of the balance between exocyst complexes and T cell signaling pathways may be an important reason for the progression of CC (45). In this study, we did not find that the *EXOC1* gene rs13117307 SNP is associated with CC susceptibility. In the stratified study, we found that alleles T of rs13117307 when SCC-Ag is greater than 1.5 ng/ml will increase the risk of CC. However after adjusting for factors such as age, height, and weight, the correlation disappeared. Rs13117307 SNP was not associated with CC age, menarche age, menopausal status, and tumor size. A genome-wide correlation study based on the Chinese population by Shi et al. showed that the rs13117307 SNP located in the *EXOC1* gene is significantly associated with cervical cancer susceptibility (45). The study by Sebastian Łaźniak et al. confirmed that the rs13117307 SNP variant may up-regulate EXCO1 transcription and is involved in the occurrence and spread of cervical squamous cell carcinoma in Poland (46).

The role of BCL2 in the development of tumors is still unclear. BCL2 was originally considered to be an anti-apoptotic regulatory protein (47), but it also has the effect of inhibiting proliferation (21). Therefore, BCL2 has both carcinogenic and tumor suppressive effects. This also explains why the prognostic significance of BCL2 expression depends on the type of cancer. Studies have shown that HPV E6 oncogenic protein is related to a variety of apoptosis regulators, such as BCL2 (48, 49). In the unit point analysis of this study, the rs2270115 located in the BCL2 gene did not show a significant association with CC. In the stratified study, it was found that compared with rs2270115 wild genotype CC, the genotype AA was association with CC when SCC-Ag is greater than 1.5 ng/ml. Fernandes et al. published a study on BCL2 gene polymorphism and cervical intraepithelial neoplasia (CIN), and found that rs2279115 SNP is not significantly related to CIN, but confirmed that the promoter SNP rs2279115 is related to elevated BCL2 protein expression (50). In addition, Ahmed HS and Qiu XG et al. found that rs2279115 SNP can increase the risk of HCV-related hepatocellular carcinoma and glioma (51, 52). Bozovic-Spasojevic found that high expression of BCL2 is associated with a good prognosis of breast cancer (53). Therefore, whether the BCL2 gene polymorphism is related to the risk of CC or cervical precancerous lesions still needs a large sample size and multi-center further research.

The lncRNAs of CCAT2 contain the transcript of rs6983267 SNP (54), which is located at 335kb downstream of MYC proto-oncogene (55). MYC oncogene is a target gene of Wnt signaling pathway, which is constitutively activated in the early development of various cancers including CC. Previous articles showed that the rs6983267 SNP located in the MYC enhancer region is related to the susceptibility of various cancers (30–34, 56, 57). However, it is controversial whether there is a correlation between SNP rs6983267 and the proto-oncogene MYC (58, 59). In this study, we did not find that rs6983267 SNP is related to the risk of CC. However, compared with rs6983267 wild genotype GG, genotype TG/TG+TT may increase the risk of CC in premenopausal women. The report by Sebastian Łaźniak et al. showed that CCAT2 rs6983267 SNP can contribute to the increase of MYC expression and the development and spread of cervical squamous cell carcinoma (29). The difference in genetic background and living environment may be the reason why this study is inconsistent with the report of Sebastian Łaźniak et al.

CARD8, a member of the caspase recruitment domain family, is an important part of the inflammasome. It can affect protein-protein, apoptosis, caspase-1 activity, regulate caspase-1-mediated activation of IL-1 and IL-8, and activate kappa -B (NFkB) (27, 28). NFkB is a key regulator of genes and tumorigenesis. Studies have reported that human papillomavirus 16 E5 tumor protein can up-regulate COX-2 expression through NFkB and mediate the occurrence of CC (60). The Rs7248320 SNP is located in the upstream region of CARD8 gene, which may affect the normal expression of CARD8. CARD8 may affect the progress of tumor biology by inhibiting cell apoptosis and participating in the NFkB signaling pathway. In this study, we did not find a significant association between the CARD8 gene rs7248320 SNP and the risk of CC in Heilongjiang Province, China. In further stratification studies, it still did not show significant statistical significance. However, Jian Yin et al. reported that rs7248320 SNP can increase the risk of CC, especially for premenopausal women (61). Compared with the study of Jian Yin et al., the sample size of this study is relatively small, so we will further expand the sample size to improve this study.

The occurrence and development of CC is a long-term, continuous, multi-factor, multi-step process. In order to evaluate the impact of multi-gene loci on CC, we conducted a haplotype study, which showed that there was no significant difference between CC and healthy controls.

This study was the first to analyze the gene polymorphisms of *EXCO1*, *BCL2*, *CCAT2* and *CARD8* in women with CC in the northern Chinese Han population. Successfully confirmed that *EXCO1*, *BCL2* and *CCAT2* gene polymorphisms may be involved in the occurrence and development of CC. This discovery gives us a deeper understanding of the incidence of CC and provides new ideas for the treatment of CC in the future.

## Data Availability Statement

The datasets presented in this study can be found in online repositories. The names of the repository/repositories and accession number(s) can be found below: https://figshare.com/s/227c1afeffd8d6240684.

## Ethics Statement

The studies involving human participants were reviewed and approved by the Ethics Committee of the Second Affiliated Hospital of Harbin Medical University. Written informed consent to participate in this study was provided by the participants’ legal guardian/next of kin. Written informed consent was obtained from the individual(s), and minor(s)’ legal guardian/next of kin, for the publication of any potentially identifiable images or data included in this article.

## Author Contributions

YF: data collection, data analysis, and manuscript writing. ZW: language modification and data analysis in the revised manuscript. MZ and SL: correction of the revised manuscript. TJ, SD, and LG: data collection and reviewing of relevant literature. XL and SZ: data collection and data analysis. XK: collecting and analyzing of patient information and data. JT: correction in the revised manuscript, LS: experimental design and overall planning. All of the authors contributed to the article and approved the submitted version.

## Funding

This study was supported by the National Natural Science Foundation of China (No. 82071929).

## Conflict of Interest

The authors declare that the research was conducted in the absence of any commercial or financial relationships that could be construed as a potential conflict of interest.

## Publisher’s Note

All claims expressed in this article are solely those of the authors and do not necessarily represent those of their affiliated organizations, or those of the publisher, the editors and the reviewers. Any product that may be evaluated in this article, or claim that may be made by its manufacturer, is not guaranteed or endorsed by the publisher.
